# BKS-112, a Selective Histone Deacetylase 6 Inhibitor, Suppresses Triple-Negative Breast Cancer Cells via AKT/mTOR Pathway

**DOI:** 10.3390/antiox14111291

**Published:** 2025-10-28

**Authors:** Sreevarsha Gali, Swati Sharma, Hyunji Noh, In Su Kim, Hyung Sik Kim

**Affiliations:** 1 School of Pharmacy, Sungkyunkwan University, 2066, Seobu-ro, Jangan-gu, Suwon 16419, Republic of Korea; sreevarsha@skku.edu (S.G.); sharmaswati2611@gmail.com (S.S.); insukim@skku.edu (I.S.K.); 2 College of Medicine, Sungkyunkwan University, 2066, Seobu-ro, Jangan-gu, Suwon 16419, Republic of Korea

**Keywords:** TNBC, HDAC6, BKS-112, reactive oxygen species, apoptosis, autophagy

## Abstract

Triple-negative breast cancer (TNBC) remains a leading cause of cancer-related mortality in women, characterized by its aggressive nature and limited therapeutic options. TNBC is defined by the absence of estrogen receptor (ER), progesterone receptor (PR), and human epidermal growth factor receptor 2 (HER2) expression, which excludes patients from targeted endocrine and HER2-directed therapies, contributing to poor prognosis. This study investigates BKS-112, a potent histone deacetylase 6 (HDAC6) inhibitor, for its anticancer activity against TNBC using MDA-MB-231 cells. We assessed HDAC protein expression and their prognostic implications, alongside in vitro experiments analyzing cell viability, apoptosis, autophagy, and colony formation. BKS-112 exhibited dose- and time-dependent reductions in cell viability, significant morphological alterations, and decreased colony formation. The compound increased the acetylation of histones H3, H4, and α-tubulin while downregulating HDAC6 expression and activity. Additionally, BKS-112 reduced cell migration, demonstrating anti-metastatic potential. It induced G1 phase cell cycle arrest and modulated key regulators, including cyclins and cyclin-dependent kinases (CDKs). Apoptosis was promoted through mitochondrial pathways, evidenced by changes in Bcl-2, Bax, and caspase activation. BKS-112 also elevated reactive oxygen species (ROS) levels, affecting apoptosis-related PI3K/AKT signaling. Autophagy was triggered by upregulating LC3 and Atg-7 expression. Collectively, these findings suggest that BKS-112 exerts robust anticancer effects by inducing cell cycle arrest, apoptosis, and autophagy, highlighting its therapeutic promise for TNBC treatment.

## 1. Introduction

Breast cancer remains a leading cause of cancer-related mortality among women, accounting for 25% (1.7 million) of all cases globally and resulting in a death every 15 min [1]. In the United States, breast cancer was responsible for 1200 deaths among women aged 20 to 39 years and 9727 deaths among those aged 40 to 59 years in 2019. Since the mid-2000s, the incidence of breast cancer has shown a consistent annual increase of approximately 0.5% [2,3]. Addressing breast cancer on a global scale necessitates the development of innovative approaches and novel treatments. Breast cancer classification, based on cellular markers that inform targeted therapies, comprises three major groups: (a) estrogen receptor (ER) or progesterone receptor (PR) positive, (b) human epidermal growth factor receptor 2 (HER2, encoded by the ERBB2 gene) positive, with or without ER and PR expression, and (c) triple-negative breast cancer (TNBC), which is defined by the lack of ER and PR expression and absence of HER2 amplification [4,5].

This subset of invasive breast cancers, comprising 10–20% of cases, is highly aggressive, has limited effective therapeutic options [6], and is associated with poor prognosis and survival outcomes [4,7]. Unlike patients with estrogen receptor-positive or HER2-positive tumors, who benefit from targeted therapies, those with TNBC have few targeted treatment options available [4,5]. The absence of these molecular targets necessitates the development of novel therapeutic approaches, such as targeting epigenetic regulators like HDAC6. Research into TNBC has explored various molecular mechanisms, including the roles of histone deacetylases (HDACs), androgen receptor (AR), vascular endothelial growth factor (VEGF), impaired DNA repair mechanisms, the mammalian target of rapamycin (mTOR), and the Src oncogene pathway [8].

Human HDACs consist of 18 enzymes grouped into four classes. Classes I, II, and IV are zinc-dependent, while class III relies on NAD+ for activity. Class I includes HDACs 1, 2, 3, and 8; Class IIa comprises HDACs 4, 5, 7, and 9; Class IIb includes HDACs 6 and 10; and Class IV consists of HDAC 11. HDAC6, a unique zinc-dependent histone deacetylase in class IIb, is mainly located in the cytoplasm and is distinguished by its multifunctional nature. It possesses two distinct catalytic domains, setting it apart from other HDACs [9,10]. It regulates the acetylation of non-histone proteins, such as cortactin and α-tubulin, and modulates the chaperone activity of heat shock protein 90 (HSP90) [11,12]. HDAC6 is overexpressed in several cancers such as breast cancer [13], pancreatic cancer [12], prostate cancer, ovarian cancer, myeloid leukemia [10], B and T cell lymphomas [14], glioblastoma [15], oral squamous cell carcinoma [16], bladder cancer, and lung cancer [12].

HDAC6 plays a multifaceted role in regulating several cellular processes, including cell cycle progression, proliferation, migration, protein trafficking and degradation, cell shape modulation [17], as well as autophagy, apoptosis, and chemotherapy sensitivity in various cancers [18,19]. Recent findings underscore the need for carefully chosen targeted drugs that inhibit HDAC6, as this selectivity could be pivotal for improving anticancer treatment strategies [10,15]. In breast cancer models, HDAC6 is implicated in promoting metastatic dissemination and enhancing the motility of cancer cells [20,21]. Previous studies have demonstrated that MDA-MB-231 cells exhibit significantly higher levels of HDAC6 expression compared to MCF-7 cells [22]. However, further research is needed to explore the differential roles of HDAC6 in various breast cancer subtypes and to determine the therapeutic potential of selective HDAC6 inhibitors.

In this study, we synthesized BKS-112, a chemical compound described as (E)-N-hydroxy-3-(2-(4-fluorostyryl) thiazol-4-yl) propanamide, as a potent HDAC6 inhibitor. We began with preliminary molecular docking analyses using HDAC6 (PDB: 5EDU) and HDAC1 (PDB: 4BKX) to assess the binding potential of our designed derivatives. Based on these computational insights, we synthesized the derivatives and performed in vitro enzymatic assays to evaluate HDAC1 and HDAC6 inhibition [23]. This comprehensive approach led to the identification of BKS-112 as a highly potent and selective HDAC6 inhibitor, characterized by improved safety and cellular selectivity. Notably, the incorporation of a phenylethene cap in BKS-112 significantly enhanced HDAC6 selectivity, highlighting the importance of rigidifying the cap group to optimize specificity toward HDAC subtypes [23].

This study aimed to advance the understanding and development of novel therapeutic agents for the precise treatment of TNBC. By investigating new compounds and elucidating their mechanisms of action, we sought to address the challenges posed by drug resistance and identify more effective clinical targets. Our findings aim to pave the way for improved treatment strategies and enhanced outcomes for patients with TNBC.

## 2. Materials and Methods

### 2.1. Compounds

BKS-112 (6u, denoted as (E)-N-hydroxy-3-(2-(4-fluorostyryl) thiazol-4-yl) propanamide) was obtained from Professor Young Hoon Jung (Department of Pharmacy, Sungkyunkwan University, Suwon, Republic of Korea) [23]. SAHA was purchased from Sigma-Aldrich (St. Louis, MO, USA). Solutions of BKS-112 (50 mM) and SAHA (5 mM) were prepared in dimethyl sulfoxide (DMSO).

### 2.2. Reagents

Dulbecco’s modified Eagle’s medium (DMEM) was purchased from WELGENE Inc. (Gyeongsangbuk-do, Republic of Korea), and fetal bovine serum (FBS), Dulbecco’s phosphate-buffered saline (DPBS), and trypsin were obtained from Gibco Invitrogen Corporation (Carlsbad, CA, USA). MTT, Pierce bicinchoninic acid (BCA) protein assay reagents A and B, and 4′,6-diamidino-2-phenylindole (DAPI) were obtained from Thermo Fisher Scientific (Invitrogen, Grand Island, NY, USA). The FITC Annexin V apoptosis detection kit was purchased from BD Pharmingen (Franklin Lakes, NJ, USA). Acridine orange, crystal violet, and Triton X-100 were purchased from Sigma-Aldrich (St. Louis, MO, USA). Bovine serum albumin was purchased from Bovogen Biologicals Pty Ltd. (East Keilor, VIC 3033, Australia). Luminata™ Crescendo Western Horseradish Peroxidase (HRP) Substrate and polyvinylidene difluoride membrane were purchased from Merck Millipore Korea (Seoul, Republic of Korea).

### 2.3. Antibodies

Primary antibodies against HDAC1 (ab7028-50), HDAC2 (ab219053), HDAC3 (ab7030-50), HDAC4 (#2072), HDAC5 (sc-133225), HDAC6 (ab133493), HDAC7 (sc-74563), acetyl histone H3 (#9677), acetyl histone H4 (sc-8660-R), α-tubulin (sc-8035), acetyl α-tubulin (#5335), tissue inhibitors of metalloproteinase (TIMP) 1 (ab1827), TIMP 2 (sc-5539), metalloproteinase (MMP) 2 (sc-13595), MMP 9 (sc-10737), Cyclin A (sc-751), Cyclin B1 (#4138), Cyclin D1 (sc-718), Cyclin E (sc-481), cyclin-dependent kinase (CDK) 1 (ab133327), CDK 2 (ab7954-1), CDK 4 (sc-260), CDK 6 (sc-7181), P21 (sc-6246), P27 (sc-528), P16 (sc-468), B-cell lymphoma 2 (Bcl2)-associated protein x (Bax; ab32503), Bcl 2 (ab32124), Caspase 3 (#9662s), Cleaved caspase 3 (#9661), Caspase 8 (#4790), Caspase 9 (#9508), P53 (#2527), Phospho P53 (#9286), cytochrome C (sc-7159), poly-ADP ribose polymerase (PARP; sc-7150), Cleaved PARP (#5625S), light chain 3 (LC3; ab51520), P62 (ab56416), autophagy-related gene (ATG) 7 (#2631), ATG 5 (#12994), Beclin 1 (#3738S), mTOR (#2983S), Phospho-mTOR (#2971S), adenosine monophosphate-activated protein kinase (AMPK; 2532), Phospho AMPK (2535), extracellular signal-regulated kinase (ERK; A4782), Phospho ERK (sc-7383), P38 (#9212), Jun N-terminal kinase (JNK; #9252), phosphoinositide 3-kinase (PI3K; #4292S), Phospho PI3K (#4228), AKT (#9272), Phospho AKT (#9271S), and β-Actin (sc-8432). The HRP-conjugated secondary antibodies were purchased from Santa Cruz Biotechnology (Santa Cruz, CA, USA).

### 2.4. Cell Culture Condition and Viability Assay

The American Type Culture Collection (Manassas, VA, USA) supplied the human TNBC cell line MDA-MB 231. These cells were cultured in DMEM supplemented with 10% FBS and 1% AA, following standard incubation conditions at 37 °C in a humidified atmosphere containing 5% CO_2_. The MTT assay was used to assess the metabolic activity, proliferation, cell viability, and cytotoxicity of MDA-MB 231 cells. Following 24 h after 5 × 10^4^ cells per well were seeded into 96-well plates. The cells were subjected to BKS-112 treatment at concentrations of 2, 10, and 50 µM, along with SAHA (5 µM) as the standard control. After treatment, 100 µL of MTT reagent was added and incubated at 37 °C for 3 h, followed by a 30-min incubation with DMSO. Finally, the absorbance was measured at 540 nm using a VERSA Max Microplate Reader (Molecular Devices Corp., San Jose, CA, USA).

### 2.5. Colony Formation Assay

The colony formation assay is an in vitro cell survival experiment that assesses the capacity of individual cells to form colonies. In this assay, 1 × 10^3^ cells were seeded per well in 6-well plates and incubated for 24 h. Subsequently, the cells were subjected to treatment with a BKS-112 (at concentrations of 2, 10, and 50 µM) along with SAHA (5 µM) as the standard control, and this treatment continued for two weeks. After treatment, the cells were washed twice, fixed with 4% paraformaldehyde, and stained with crystal violet. Finally, the stained colonies were imaged and analyzed using an image analyzer.

### 2.6. HDAC6 Activity Assay

The HDAC6 Activity Assay Kit Cat. No. ab284549), enables the evaluation of HDAC6 deacetylase activity by utilizing a synthetic acetylated peptide substrate that generates an AFC fluorophore that is easily detectable using a standard microplate reader. To perform the assay, cells were initially cultured and treated with varying concentrations of BKS-112 (2, 10, and 50 µM) and SAHA (used as a standard control at 5 µM). Subsequently, a lysis buffer was added, followed by centrifugation at 16,000× *g* for 10 min at 4 °C to collect the lysate supernatant. After BCA protein analysis, an appropriate amount of lysate was transferred into a 96-well white plate. An HDAC6 inhibitor control, tubacin (2 µL of 10X Tubacin), was introduced and incubated at 37 °C for 10 min. Following this, a positive control (ranging from 25 to 50 µL) was added, and the sample and positive control volumes were adjusted to 50 μL per well using HDAC6 Assay Buffer. The substrate mix (50 μL) was then added and thoroughly mixed, and the plate was sealed for a 30-min incubation at 37 °C. To halt the reaction, 10 μL of a developer was added to each well and further incubated for 10 min at 37 °C. Finally, fluorescence measurements were performed in the endpoint mode with excitation/emission wavelengths of 380/490 nm.

### 2.7. Immunocytochemistry

MDA-MB 231 cells were cultured in confocal dishes to provide a controlled environment for subsequent treatment. Cells were exposed to varying concentrations of BKS-112. After 48 h, the cells were fixed with acetone for 20 min, and excess fixative was removed using ice-cold phosphate-buffered saline (PBS). To prepare the cells for immunofluorescence analysis, they were treated with 10% normal goat serum in PBS for 30 min. Subsequently, the cells were incubated with primary antibody targeting HDAC6 (ab133493, 1:400 dilution) at 35 °C for 3 h. After washing with PBS, cells were incubated with Alexa Fluor 488-conjugated goat anti-rabbit IgG secondary for 30 min at room temperature in the dark. Cells were then counterstained with 0.1 μg/mL DAPI in PBS for 5 min to visualize nuclei. After final PBS washes, the treated and stained cells were analyzed using a K1-fluo fluorescence microscope (Nanoscope Systems, Daejeon, Republic of Korea) at 400× magnification. HDAC6 immunofluorescence was detected in the green channel (excitation ~488 nm/emission ~520 nm for Alexa Fluor 488), and DAPI nuclear staining was detected in the blue channel (excitation 405 nm/emission ~460 nm). The treated and stained cells were analyzed using a K1-fluo microscope (Nanoscope Systems, Daejeon, Republic of Korea) at 400× magnification.

### 2.8. Western Blot Analysis

Western blotting is a laboratory technique that employs gel electrophoresis to separate and identify proteins based on their molecular weight. In this experiment, MDA-MB 231 cells were cultured and treated with a BKS-112 at concentrations of 2, 10, and 50 µM and with SAHA at 5 µM. After 48 h of treatment, the cells were collected and lysed using PRO-PREP cell lysis buffer, and the protein content in the lysates was quantified using a BCA protein assay. Quantified lysates were loaded onto sodium dodecyl-sulfate-polyacrylamide gel electrophoresis gels and subsequently transferred onto nitrocellulose membranes. The membranes were blocked and subjected to immunoblotting using specific primary antibodies and HRP-conjugated secondary antibodies. Finally, the protein bands were visualized using the Luminata™ Crescendo Western HRP Substrate and captured with a ChemiDoc imaging system (Bio-Rad, Hercules, CA, USA).

### 2.9. Wound-Healing Assay

A wound-healing assay is a method employed to investigate cell migration and cell-to-cell interactions. In this study, 2 × 10^4^ cells were seeded per well in 96-well plates and allowed to grow for 24 h until the confluence exceeded 95%. Wounds were then created using a Wound Maker (Essen Bioscience, Ann Arbor, MI, USA) and subsequently treated with a BKS-112 at concentrations of 2, 10, and 50 µM, in addition to SAHA at 5 µM. Images of the wounded areas were captured at 6-h intervals, and the relative wound-healing density was quantified using IncuCyte^®^ ZOOM 2016B software (Essen Bioscience, Ann Arbor, MI, USA).

### 2.10. Cell Cycle Analysis

Flow cytometry is a powerful technique capable of detecting cell populations at various phases of the cell cycle (G0/G1, S, and G2/M) and identifying apoptotic cells (sub-G0). For this experiment, 4 × 10^4^ cells per well were cultured in a 60 × 15 mm dish and treated with BKS-112 at concentrations of 2, 10, and 50 µM in addition to SAHA at 5 µM. The treated cells were then harvested, washed with cold DPBS, and subsequently fixed with ice-cold 70% pure ethanol overnight at 4 °C. Following fixation, the cells were stained with a propidium iodide staining solution (10 µL/mL) combined with RNAase (5 µg/mL) in cold DPBS, and the cell suspension was incubated for 30 min at 37 °C in the dark. Flow cytometry analysis was performed using the Guava^®^ EasyCyte flow cytometer (Merck Millipore, Burlington, MA, USA).

### 2.11. Apoptotic Flow Cytometry Analysis

Apoptosis, a process initiated by the binding of annexin V to phosphatidylserine residues on the cell membrane, is commonly examined using flow cytometry. In this study, 4 × 10^4^ cells were seeded per well in a 60 × 15 mm dish and allowed to grow for 24 h. Subsequently, the cells were treated with a BKS-112 at concentrations of 2, 10, and 50 µM, along with SAHA at 5 µM. After this treatment period, the entire cell population, including both live and dead cells, was harvested for flow cytometry analysis employing the BD Pharmingen™ FITC Annexin V Apoptosis Detection Kit (BD Biosciences, San Diego, CA, USA). The harvested cells were stained with annexin V Recom FITC (2 µL) and propidium iodide staining solution (4 µL), which were diluted with annexin V 1X binding buffer (100 µL), and the staining was conducted for 30 min in the dark. Subsequently, the stained cells were suitably diluted with 1X binding buffer and analyzed using flow cytometry (Guava EasyCyte flow cytometer; Merck Millipore, Burlington, MA, USA).

### 2.12. DAPI Staining

DAPI staining was used to evaluate the overall cell morphology, determine the number of nuclei, and identify apoptotic nuclei characterized by fragmented or condensed chromatin. First, 1 × 10^3^ cells/well were seeded into a confocal dish and allowed to grow for 24 h. Subsequently, the cells were treated with BKS-112 at concentrations of 2, 10, and 50 µM, along with SAHA at 5 µM. The dishes were then rinsed twice with DPBS, and a 300 nM DAPI staining solution was added to the cells. The staining solution was incubated for 1–5 min in the dark at room temperature. Following the DAPI staining period, the DAPI solution was removed, and the cells were washed with DPBS five times to eliminate any debris. Apoptotic bodies and nuclear morphology within the cells were analyzed using a K1-fluo microscope at 400× magnification (Nanoscope Systems, Daejeon, Republic of Korea).

### 2.13. Determination of Intracellular Reactive Oxygen Species

MDA-MB-231 cells were seeded into a 96-well black plate and allowed to incubate for 24 h. The cells were then treated with BKS-112 at concentrations of 2, 10, and 50 µM, alongside SAHA (5 µM) as a reference compound. After a 48-h incubation period, the media were removed, and the wells were washed with Dulbecco’s phosphate-buffered saline (DPBS). To assess reactive oxygen species (ROS) generation, 20 µM of 2′,7′-dichlorofluorescin diacetate (DCFDA) was added to each well (100 µL per well, excluding black wells). For the black wells, 100 µL of FBS buffer was added instead, and the cells were incubated for an additional 45 min. Finally, 100 µL of the positive control was added to the designated wells, and fluorescence was measured using the PerkinElmer Multilabel Plate Reader_EnVision™ (DawinBio, Gyeonggi-do, Republic of Korea).

### 2.14. Autophagy Assay

In this study, 4 × 10^4^ cells were seeded per well in a 60 × 15 mm dish and allowed to grow for 24 h. Subsequently, the cells were treated with BKS-112 at concentrations of 2, 10, and 50 µM, along with SAHA at 5 µM for 48 h. The cells were stained with a rhodamine solution (0.5 µM), which was diluted with DPBS, and the staining was conducted for 3 h in the dark. After this staining period, the entire cell population, including both live and dead cells, was harvested for flow cytometry. Subsequently, the stained cells were suitably diluted with DPBS and analyzed using flow cytometry (Guava EasyCyte flow cytometer; Merck Millipore, Burlington, MA, USA).

Acridine orange is a fluorescent dye known to emit distinct colors and selectively bind to DNA or RNA in cells, aiding in the differentiation of cellular organelles. For the staining procedure, 1 × 10^3^ cells were seeded per well in a confocal dish and incubated for 24 h. Subsequently, the cells underwent treatment with BKS-112 at concentrations of 2, 10, and 50 µM and SAHA at 5 µM, serving as the standard control, for 48 h. Following treatment, the cells were rinsed with DPBS and stained with acridine orange (5 mg/mL) for 15 min. The stained cells were thoroughly washed five times with DPBS to eliminate excess dye. Acridine orange fluorescence was detected using dual-wavelength emission: green fluorescence (excitation 488 nm, emission 530 nm) for cytoplasm and nuclei, and red fluorescence (excitation 488 nm, emission > 600 nm) for acidic vesicular organelles including autophagosomes and lysosomes. Subsequently, the cells were examined under a K1-fluo microscope at 400× magnification (Nanoscope Systems, Daejeon, Republic of Korea).

Rhodamine 123 is a fluorescent dye with a strong affinity for mitochondria in living cells, making it useful for evaluating the mitochondrial membrane potential. In our experiment, cells were cultured and treated with BKS-112 alongside SAHA (used at 5 µM as the standard control) within a 6-well plate. Subsequently, a solution containing rhodamine 123 (0.5 mM) was prepared by adding it to 2 mL of DMEM and evenly distributing it across the cells in a 6-well plate. The plate was covered with foil and incubated for 3 h. After incubation, the cells were harvested, their pellet was washed with DPBS, and 1 mL of cold DPBS was added for subsequent flow cytometry analysis. Flow cytometry was used to examine and analyze the rhodamine-stained cells using a Guava EasyCyte flow cytometer (Merck Millipore, Burlington, MA, USA).

### 2.15. Statistical Analysis

The data in our study are presented as the mean value along with the standard deviation (mean ± SD). To assess the statistical differences among various groups, we employed analysis of variance (ANOVA), followed by Tukey’s multiple comparison tests. For all statistical assessments, we used a significance level of 5% (*p* < 0.05). Statistical analysis was performed using values representing the mean ± standard deviation (*** *p* < 0.001, ** *p* < 0.01, and * *p* < 0.05) using the GraphPad Prism Software (version 5.0; GraphPad Software, San Diego, CA, USA).

## 3. Results

### 3.1. Effects of BKS-112 on Cell Viability and Metastasis in MDA-MB-231 Cells

**Figure 1:**
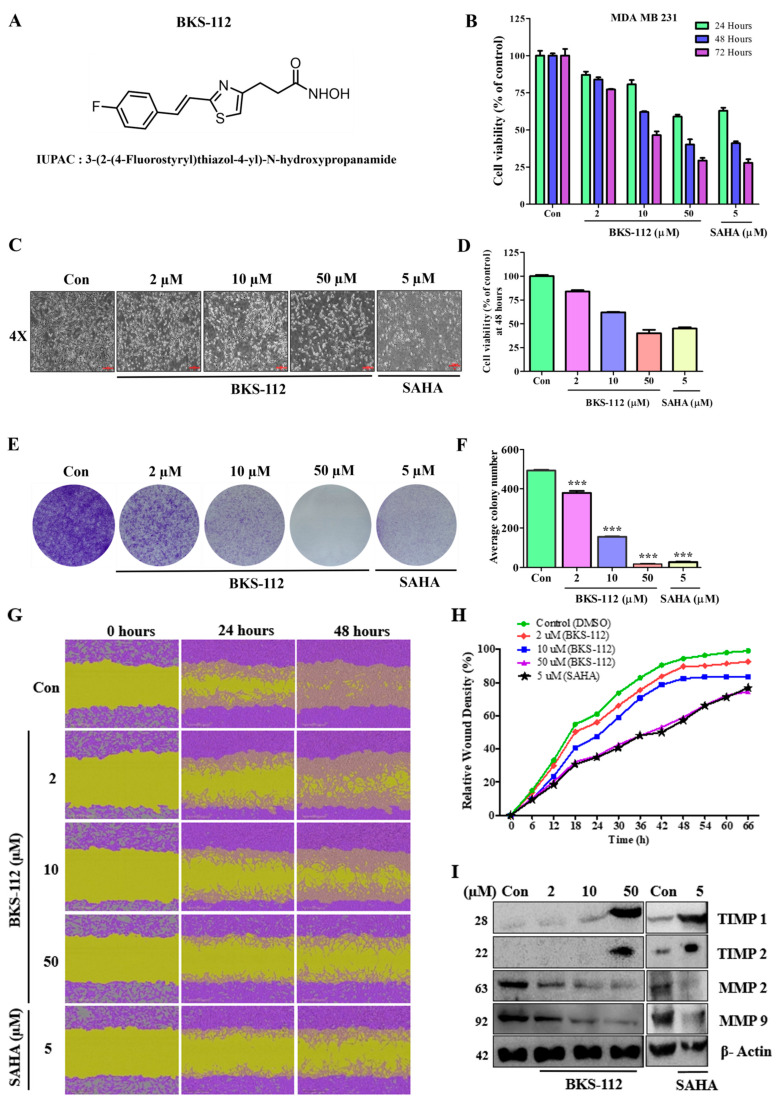
Dose-dependent modulation of cell viability and metastasis in MDA-MB-231 cells induced by BKS-112. (**A**) Chemical structure of BKS-112. (**B**) The effects of BKS-112 and SAHA on cell viability in MDA-MB-231 cells over 24, 48, and 72 h, assessed using the MTT assay. (**C**) Morphological changes in MDA-MB-231 cells observed under a light microscope after 48 h of treatment with BKS-112 and SAHA. Cells were observed using an inverted microscope at ×4 magnification. (**D**) MTT assay results showing cell viability following 48-h exposure to BKS-112. (**E**) Quantification of colony areas using an image analyzer, with representative images and quantitative data provided. (**F**) Graphical representation of colony formation, illustrating the average colony formation in MDA-MB-231 cells. Statistical analysis is presented as mean ± standard deviation (*** *p* < 0.001). (**G**) Assessment of BKS-112’s effects on cell migration and wound healing using IncuCyte software, with the wounded area depicted in green. (**H**) Graphical representation of changes in relative wound density following treatment with BKS-112 and SAHA. (**I**) Western blot analysis of protein levels for metastatic markers, including MMP-2, MMP-9, TIMP-1, and TIMP-2, in MDA-MB-231 cells after BKS-112 and SAHA treatment. β-Actin was used as the loading control. Supplementary Figure S1. MTT assay showing dose-dependent cytotoxicity in MCF-7 cells, Supplementary Figure S2. In SKOV3 (ovarian cancer) cells and Supplementary Figure S3. in MCF-10A (normal breast epithelial) cells after BKS-112 treatment for 24 h, demonstrating minimal cytotoxicity compared with cancer cells. Supplementary Figure S4a,b. Western blot analysis showing HDAC6 expression levels in various cancer cell lines (A549, DU145, Caki-1, SKOV3, MDA-MB-231, and MCF-7) following treatment with BKS-112 at indicated concentrations. β-actin was used as the loading control.

The chemical structure of BKS-112, identified by its IUPAC name 3-(2-(4-Fluorostyryl) thiazol-4-yl)-N-hydroxypropanamide, is depicted in Figure 1A. To evaluate the effect of BKS-112 on the growth and viability of MDA-MB-231 cells, we employed the MTT assay. Cells were cultured for 24 h and then treated with a control (DMSO) or various concentrations of BKS-112 (2, 10, and 50 μM), alongside SAHA (5 μM) as a reference. The results demonstrated a time-dependent (24, 48, and 72 h) and dose-dependent (2, 10, and 50 μM) reduction in cell growth and viability upon BKS-112 treatment compared to control cells. These findings suggest that the selective inhibition of HDAC6 by BKS-112 significantly impairs the growth and viability of MDA-MB-231 cells. The effects of BKS-112 were contrasted with those of SAHA, as cells initially cultured for 24 h were treated with DMSO, BKS-112 at various concentrations, and SAHA (5 μM). Our data indicates that the IC_50_ values of both BKS-112 and SAHA were influenced by the duration and concentration of the treatment, illustrating the dose- and time-dependent efficacy of BKS-112 in reducing MDA-MB-231 cell viability, as presented in Figure 1B. For the confirmation, supporting for MDA MB 231 cells specifically, we performed MTT cell viability assays in MCF-7 (ER-positive breast cancer), SKOV3 (ovarian cancer), and MCF-10A (normal breast epithelial) cells, as presented in  Supplementary Figures S1–S3. These results demonstrate that BKS-112 exhibits selective cytotoxicity toward cancer cells while showing significantly reduced toxicity in MCF-10A normal cells, indicating a favorable therapeutic aspect.

Additionally, we conducted Western blot analyses to assess HDAC6 expression and the inhibitory effects of BKS-112 across a diverse panel of cancer cell lines, including A549 (lung cancer), DU145 (prostate cancer), Caki-1 (renal cell carcinoma), SKOV3 (ovarian cancer), MDA-MB-231 (TNBC), and MCF-7 (ER-positive breast cancer), as shown in Supplementary Figure S4a,b. These data reveal that while HDAC6 baseline expression varies among cancer types, BKS-112 consistently reduces HDAC6 expression. Notably, MDA-MB-231 cells displayed markedly decreased HDAC6 expression upon treatment, consistent with literature reports that TNBC exhibits elevated HDAC6 levels relative to other breast cancer subtypes.

In addition to assessing cell viability, we observed notable morphological alterations in MDA-MB-231 cells following 48-h treatments with BKS-112 and SAHA. For the BKS-112 treatment, a higher concentration of 50 μM was selected for the 48 h exposure, while SAHA, a well-established histone deacetylase inhibitor, was administered at approximately 5 μM, consistent with prior research. Morphological changes were evident in cells treated with both BKS-112 and SAHA after 48 h, as shown in Figure 1C. Detailed results of the 48 h treatments are presented in Figure 1D.

To further evaluate the anticancer activity of BKS-112, we conducted a colony formation assay. The results demonstrated a dose-dependent reduction in colony formation upon treatment with BKS-112, paralleling the effects observed with SAHA. Specifically, the average colony counts of MDA-MB-231 cells consistently decreased with higher doses of BKS-112. In contrast, SAHA treatment led to only a marginal reduction in colony numbers compared to the more pronounced effects of BKS-112. The diminishing trend in colony formation with increasing doses of BKS-112 is illustrated in Figure 1E. Additionally, a graphical representation (Figure 1F) provides a clear comparison of the dose-dependent effects of BKS-112 and SAHA on colony formation.

In our experimental approach, we conducted a wound healing assay to assess the migration capacity of MDA-MB-231 cells. The results indicated a significant reduction in the wound-healing ability of cells treated with BKS-112 compared to the control group. At 48 h post-scratch, substantial differences in wound closure were observed, demonstrating a time-dependent inhibition of cell migration with both BKS-112 and SAHA treatments, as shown in Figure 1G. The relative wound areas over time are presented in Figure 1H, illustrating a clear dose- and time-dependent reduction in cell migration. To further explore the molecular mechanisms underlying these effects, we performed Western blot analysis to assess the expression of key proteins involved in cell migration, including tissue inhibitors of metalloproteinases (TIMPs) and matrix metalloproteinases (MMPs). BKS-112 and SAHA treatments led to a significant increase in TIMP expression, while MMP expression was markedly reduced (Figure 1I). These findings suggest that BKS-112 impedes cell migration by modulating the balance between TIMPs and MMPs, thereby contributing to its anti-metastatic potential.

### 3.2. Effects of BKS-112 on HDAC Protein Expression in MDA-MB-231 Cells

**Figure 2:**
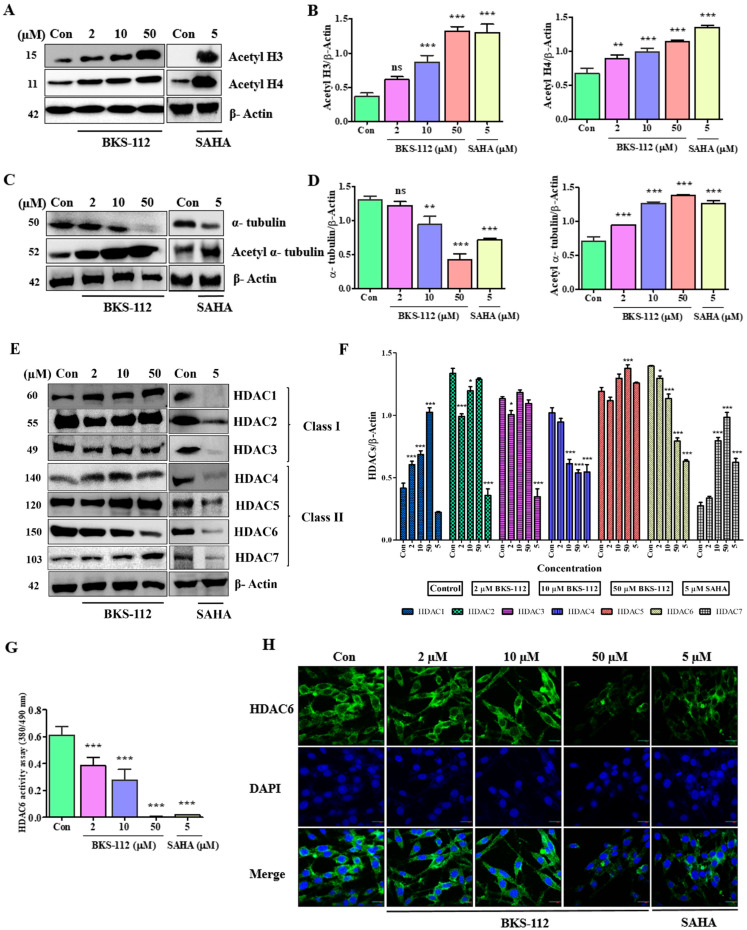
Impact of BKS-112 on histone and tubulin acetylation, and assessment of histone deacetylase (HDAC) expression. (**A**) Western blot analysis evaluating the effects of BKS-112 and SAHA on the acetylation levels of histones H3 and H4 in MDA-MB-231 cells. (**B**) Graphical representation of acetylated H3 and H4 levels observed in Western blot, with β-actin serving as the loading control. Statistical analysis is presented as mean ± standard deviation (*** *p* < 0.001, ** *p* < 0.01). (**C**) Western blot analysis assessing the effects of BKS-112 and SAHA on α-tubulin and acetylated α-tubulin protein expression levels in MDA-MB-231 cells. (**D**) Graphical representation of α-tubulin and acetylated α-tubulin levels from Western blot analysis, using β-actin as the loading control. Statistical analysis is presented as mean ± standard deviation (*** *p* < 0.001, ** *p* < 0.01). (**E**) Western blot analysis of the protein expression of HDACs (HDACs 1–7) following 48-h treatment with specified concentrations of BKS-112 and SAHA. (**F**) Graphical illustration of HDACs (HDACs 1–7) expression levels derived from Western blot analysis, with β-actin as the loading control (* *p* < 0.05, *** *p* < 0.001). (**G**) Measurement of HDAC6 enzyme activity using a fluorogenic HDAC6 assay kit to demonstrate HDAC6 selectivity. Statistical analysis is presented as mean ± standard deviation (*** *p* < 0.001). (**H**) Immunocytochemistry analysis in MDA-MB-231 cells assessing the impact of BKS-112 on intracellular HDAC6 levels. All images were captured at ×400 magnification. Fluorescence microscopy images were acquired using 40 objectives. HDAC6 was visualized with Alexa Fluor 488-conjugated secondary antibody (green channel; excitation ~488 nm), and nuclei were counterstained with DAPI (blue channel; excitation 405 nm). Images demonstrate dose-dependent reduction in HDAC6 expression following BKS-112 treatment.

Our study aimed to investigate the impact of BKS-112 on histone acetylation in MDA-MB-231 cells. We performed a detailed analysis of the expression levels of acetylated histones H3, H4, and α-tubulin using Western blot techniques. The results demonstrated a significant increase in the expression of acetylated histones in cells treated with BKS-112 (50 μM), which mirrored the effects observed with SAHA (5 μM) (Figure 2A). This elevation in histone acetylation levels suggests that BKS-112 has a strong influence on histone modification in MDA-MB-231 cells (Figure 2B,C). Furthermore, a graphical representation was provided to illustrate changes in tubulin acetylation, offering a clearer understanding of the impact on microtubule dynamics (Figure 2D). These findings emphasize BKS-112’s potential to modulate histone and tubulin acetylation, highlighting its role in altering chromatin structure and cellular dynamics in MDA-MB-231 cells.

We compared the expression levels of various HDAC proteins in MDA-MB-231 cells treated with BKS-112. The expression levels of HDACs 1, 2, and 5 remained stable, showing no significant alterations. In contrast, HDACs 3, 4, and 6 displayed a dose-dependent reduction in expression upon treatment with BKS-112. Conversely, SAHA treatment at a concentration of 5 μM led to a consistent reduction in the protein expression of HDACs 1–7, as illustrated in Figure 2E,F. These findings reveal an important distinction between BKS-112 and SAHA in their HDAC inhibition profiles. While SAHA exhibits broad pan-HDAC inhibitory activity affecting HDAC1-7 expression (Figure 2E,F), BKS-112 demonstrates a more selective pattern, preferentially targeting HDAC6 while also showing moderate activity against HDAC3 and HDAC4, and notably sparing HDAC1, HDAC2, and HDAC5. This moderately selective multi-HDAC inhibition profile distinguishes BKS-112 from both pan-HDAC inhibitors like SAHA and highly selective HDAC6 inhibitors like tubacin or ACY-1215. The preservation of HDAC1, HDAC2, and HDAC5 expression suggests that BKS-112 may offer reduced toxicity compared to pan-HDAC inhibitors while maintaining potent anticancer activity through coordinated inhibition of HDAC6, HDAC3, and HDAC4, which are critically involved in cancer cell survival, proliferation, and metastasis. These detailed findings highlight the selective regulatory effects of BKS-112 on HDAC expression, providing valuable insights into its potential therapeutic role in targeting HDACs specifically associated with triple-negative breast cancer (TNBC).

To evaluate the HDAC6 enzymatic activity of BKS-112 in MDA-MB-231 cells, we conducted dose-dependent measurements that revealed a significant reduction in HDAC6 activity corresponding to increasing concentrations of BKS-112. Notably, a comparative analysis with SAHA underscored the specificity and efficacy of BKS-112 in selectively inhibiting HDAC6, as illustrated in Figure 2G. This comparison provides crucial insights into the targeted action of BKS-112 on HDAC6. To further analyze HDAC6 expression, we performed immunocytochemistry to observe the cellular distribution and localization of HDAC6 in cultured MDA-MB-231 cells. The results demonstrated a consistent decrease in HDAC6 protein levels in cells treated with BKS-112. Confocal microscopy images were acquired using 40X objectives. HDAC6 was detected using Alexa Fluor 488 with excitation at approximately 488 nm and emission collected at 500–550 nm (green channel). Nuclei were counterstained with DAPI and visualized with excitation at 405 nm and emission collected at 420–480 nm (blue channel) for the nucleus—highlighting the impact of BKS-112 on HDAC6 expression and localization (Figure 2H). These findings emphasize the potent and selective inhibitory effects of BKS-112 on HDAC6 in MDA-MB-231 cells.

### 3.3. Effects of BKS-112 on Cell Cycle Regulation in MDA-MB-231 Cells

**Figure 3:**
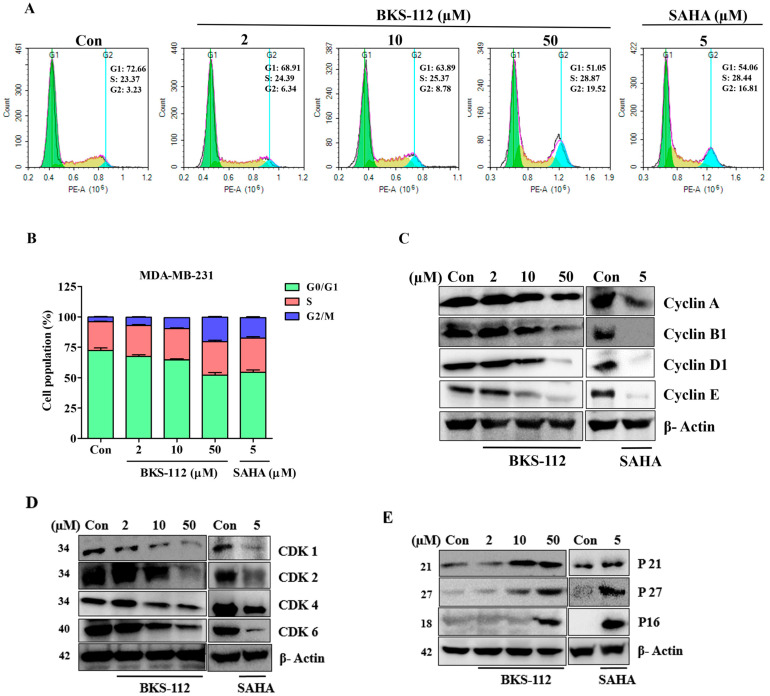
Influence of BKS-112 on cell cycle regulation in MDA-MB-231 cells. (**A**) Flow cytometry analysis illustrating the effects of BKS-112 and SAHA treatment on cell cycle distribution in MDA-MB-231 cells. (**B**) Bar graph depicting the distribution of MDA-MB-231 cells across different phases of the cell cycle following BKS-112 treatment. (**C**) Western blot analysis evaluating the effects of BKS-112 and SAHA on cyclins, including Cyclin A, Cyclin B1, Cyclin D1, and Cyclin E, in MDA-MB-231 cells, with β-actin as the loading control. (**D**) Western blot analysis assessing the impact of BKS-112 and SAHA on CDKs, specifically CDK1, CDK2, CDK4, and CDK6, in MDA-MB-231 cells, with β-actin as the loading control. (**E**) Western blot analysis examining the influence of BKS-112 and SAHA on cell cycle regulatory proteins, including p21, p27, and p16, in MDA-MB-231 cells, with β-actin as the loading control.

We utilized flow cytometry to investigate the regulation of the cell cycle in MDA-MB-231 cells following treatment with BKS-112 and SAHA. Flow cytometry analysis revealed that BKS-112 treatment significantly altered cell cycle distribution in MDA-MB-231 cells, with a dose-dependent decrease in G1 phase and corresponding increase in G2/M phase cells, indicating G1 phase arrest, as depicted in Figure 3A. A bar graph further illustrates the relative distribution of cells across different phases of the cell cycle in response to BKS-112 and SAHA treatment (Figure 3B), highlighting the dose-dependent effects on cell cycle regulation.

To further elucidate the molecular mechanisms underlying cell cycle regulation, we performed a comprehensive Western blot analysis. The results demonstrated a dose-dependent reduction in the expression of cyclins with increasing concentrations of BKS-112 and SAHA, as shown in Figure 3C. Likewise, the expression levels of cyclin-dependent kinases (CDKs) exhibited a significant decrease following treatment with BKS-112, mirroring the effects observed with SAHA in Figure 3D. Additionally, we observed upregulation of key cell cycle checkpoint regulators, including P21, P27, and P16, after treatment with both BKS-112 and SAHA (Figure 3E). These findings collectively suggest that BKS-112 exerts a pronounced inhibitory effect on the cyclin-CDK complex, highlighting the crucial role of HDAC6 in modulating cell cycle progression. This comprehensive analysis provides valuable insights into the potential of BKS-112 as an effective agent for disrupting cell cycle regulation in MDA-MB-231 cells.

### 3.4. Effects of BKS-112 on the Apoptotic Cell Death in MDA-MB-231 Cells

**Figure 4:**
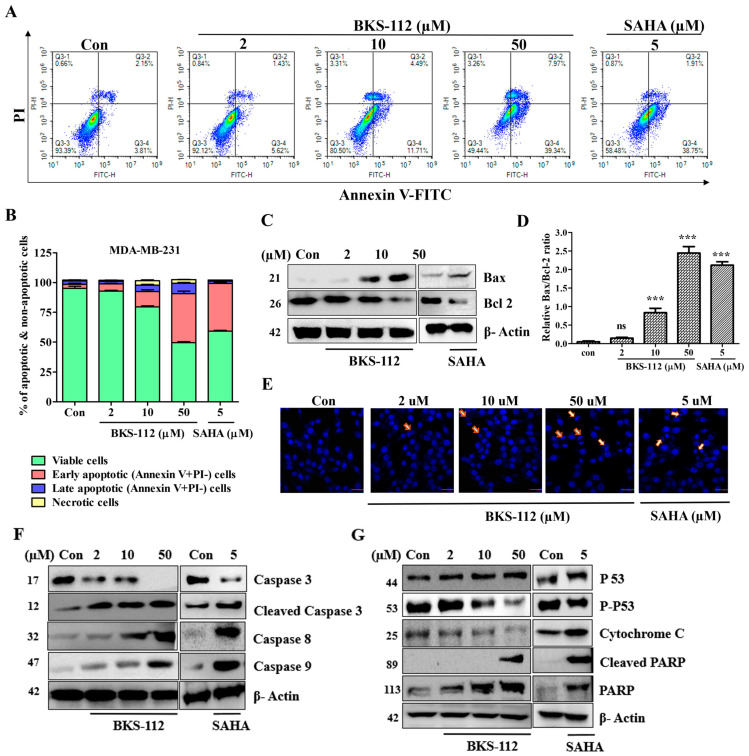
Influence of BKS-112 on apoptotic activity in MDA-MB-231 cells. (**A**) Flow cytometry analysis of MDA-MB-231 cells stained with propidium iodide and fluorescein isothiocyanate-conjugated Annexin V after 48-h exposure to BKS-112, showing the induction of apoptosis. (**B**) Bar graph depicting the extent of apoptotic cell death as determined through flow cytometry analysis. (**C**) Western blot analysis assessing the expression levels of pro-apoptotic proteins in MDA-MB-231 cells following treatment with BKS-112 and SAHA. (**D**) Graphical representation of the Bcl-2/Bax ratio, calculated from Western blot data, with β-actin serving as the loading control. (**E**) Observation of nuclear morphological changes in MDA-MB-231 cells treated with BKS-112 and SAHA using 4′,6-diamidino-2-phenylindole (DAPI) nuclear staining. Images were acquired with a confocal K1-fluo microscope (Nanoscope Systems, Daejeon, Republic of Korea) at ×400 and ×600 magnifications. DAPI fluorescence was detected using UV excitation (405 nm) and emission filter (420–480 nm, blue channel). Statistical analysis is presented as mean ± standard deviation (*** *p* < 0.001). (**F**) Western blot analysis evaluating the impact of BKS-112 and SAHA on caspases, including Caspase 3, Cleaved Caspase 3, Caspase 8, and Caspase 9, in MDA-MB-231 cells, with β-actin as the loading control. (**G**) Western blot analysis assessing the effect of BKS-112 and SAHA on apoptotic proteins, such as P53, phosphorylated P53 (p-P53), Cytochrome C, Cleaved PARP, and PARP, in MDA-MB-231 cells, with β-actin used as the loading control.

The apoptotic effects of BKS-112 were evaluated using Annexin V-FITC/PI staining assays, which revealed a significant increase in the apoptotic cell population, facilitating a comparative analysis between the control group and BKS-112 treatment, as demonstrated in Figure 4A. These results underscore the pro-apoptotic activity of BKS-112 in MDA-MB-231 cells, mirroring the effects observed with SAHA. Flow cytometry analysis provided a quantitative assessment of apoptosis at the protein level, confirming the enhanced apoptotic response in Figure 4B. In parallel, BKS-112 and SAHA treatment led to increased Bax expression levels, accompanied by a marked reduction in Bcl-2 expression, as depicted in Figure 4C. To illustrate the apoptotic balance, the ratio of Bcl-2 to Bax was calculated and graphically represented in Figure 4D.

To further confirm apoptosis, DAPI nuclear staining was utilized to visualize apoptotic nuclei, distinguished by fragmented or condensed chromatin. Following BKS-112 treatment, a prominent presence of apoptotic nuclei was observed compared to the control. Notably, SAHA exhibited a lower number of apoptotic nuclei, suggesting that BKS-112 exerts a more pronounced apoptotic effect. The nuclear morphological changes induced by BKS-112 are illustrated in Figure 4E, with images captured using a confocal K1-Fluo microscope. We further explored the complex landscape of apoptotic proteins by analyzing their expression in MDA-MB-231 cell lysates. As depicted in Figure 4F, the expression of caspase-3 was notably reduced following treatment. Extending our investigation beyond caspases, we assessed the expression levels of p53, PARP, and their cleaved forms. Our findings demonstrated an upregulation of p53 and cleaved PARP at higher doses of BKS-112 and SAHA. Interestingly, the expression patterns of phosphorylated p53 and cytochrome c exhibited a divergent response to BKS-112 treatment, with both showing a decrease, as illustrated in Figure 4G.

### 3.5. Effects of BKS-112 on Autophagy Cell Death Pathway in MDA-MB-231 Cells

**Figure 5:**
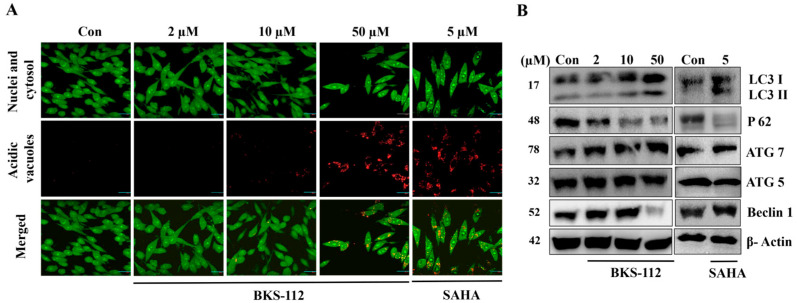
The effects of BKS-112 on autophagy-related proteins in MDA-MB-231 cells. (**A**) Representative micrographs of MDA-MB-231 cells treated with BKS-112 and SAHA, stained with acridine orange (magnification ×400) to visualize autophagic vacuoles. (**B**) Western blot analysis showing the protein levels of autophagy markers, including LC3 I, LC3 II, p62, ATG7, ATG5, and Beclin 1, in MDA-MB-231 cells following treatment with BKS-112 and SAHA. β-Actin is used as the loading control.

Autophagy activation was evident, characterized by the accumulation of lysotropic dyes in acidic organelles in a pH-dependent manner. Minimal red fluorescence was observed in the control group, indicating a lack of autophagic vacuoles. In contrast, cells treated with BKS-112 and SAHA exhibited pronounced autophagic vacuoles, with a marked increase in red fluorescence intensity at 48 h, as clearly shown in Figure 5A. These findings visually illustrate the autophagic response induced by HDAC6 inhibition and highlight the potential of BKS-112 to modulate autophagy dynamics in MDA-MB-231 cells.

We investigated the impact of BKS-112 on the expression of autophagy-related proteins through Western blot analysis. The results indicated a marked increase in LC3 (I and II) expression following treatment with both BKS-112 and SAHA, accompanied by a modest elevation in ATG7 levels. The p62 expression exhibited a dose-dependent decline with SAHA treatment, whereas ATG5 expression remained unchanged with BKS-112 and showed a slight reduction with SAHA. Regarding Beclin 1, BKS-112 treatment did not result in any significant changes compared to the control, although slight alterations were observed following SAHA treatment (Figure 5B). These findings offer valuable insights into the regulatory effects of BKS-112 on the expression of autophagy-related proteins, contributing to the broader scholarly discourse on this complex cellular process.

### 3.6. Effects of BKS-112 on Intracellular ROS Levels in MDA-MB-231 Cells

**Figure 6:**
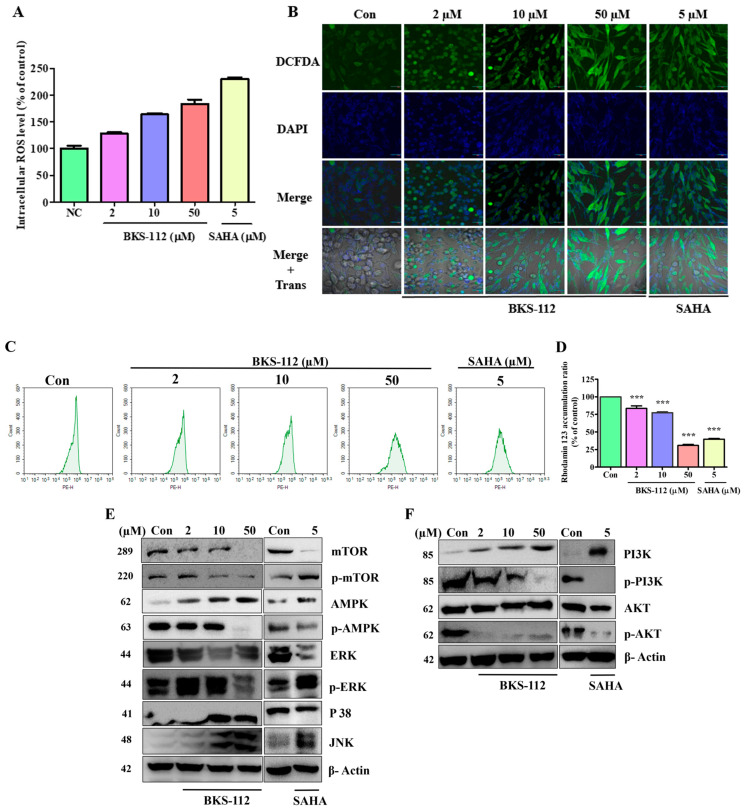
Investigation of intracellular ROS levels and the expression of proteins involved autophagy and apoptotic signaling pathways. (**A**) Evaluation of intracellular ROS production in MDA-MB-231 cells treated with BKS-112, demonstrating a dose-dependent increase compared to both the control group and SAHA-treated cells. (**B**) Microscopic images of DCFDA staining used to assess ROS expressions in cells following BKS-112 treatment. All images were captured at ×400 magnification, and scale bars represent 20 μm. (**C**) Flow cytometry analysis showing the percentage of rhodamine 123 accumulation in cells treated with BKS-112 and SAHA. (**D**) Bar graph depicting the rhodamine 123 accumulation ratio as a percentage relative to the control group. Statistical analysis is presented as mean ± standard deviation (*** *p* < 0.001). (**E**) Western blot analysis revealing the expression of proteins involved in the ROS and apoptosis-associated autophagy pathway, including mTOR, p-mTOR, AMPK, p-AMPK, ERK, p-ERK, p38, and JNK, with β-actin used as the loading control. (**F**) Western blot analysis showing the expression of proteins related to the apoptotic pathway in the context of ROS and autophagy, such as PI3K, p-PI3K, AKT, and p-AKT, with β-actin serving as the loading control.

The assessment of intracellular ROS levels is a crucial parameter for understanding the potential damage inflicted on cellular components, including DNA, RNA, and proteins, which can ultimately lead to cell death. Using the DCFDA assay, we measured intracellular ROS production in MDA-MB-231 cells following BKS-112 treatment. The results demonstrated a dose-dependent increase in ROS levels compared to both the control and SAHA treatments in Figure 6A. These findings enhance our understanding of BKS-112’s impact on cellular oxidative stress, underscoring its potential implications in MDA-MB-231 cells.

Microscopic images provide critical visual evidence of the outcomes of DCFDA staining, employed to assess ROS expression in cells treated with BKS-112. An increase in green fluorescence intensity indicated elevated ROS levels (Figure 6B). Additionally, we utilized rhodamine 123 staining to explore the interplay between ROS, apoptosis, and autophagy. Flow cytometry analysis facilitated the examination of these complex cellular interactions. The results demonstrated a dose-dependent decrease in the percentage accumulation of rhodamine 123 with increasing doses of BKS-112 and SAHA compared to the control in Figure 6C,D. These findings highlight the substantial impact of BKS-112 on ROS accumulation, exhibiting a phenomenon similar to that observed with SAHA.

To comprehensively examine ROS-related protein expressions associated with autophagy and apoptosis, we conducted Western blot analysis. Our data showed that BKS-112 treatment led to a decrease in the expression of proteins such as mTOR, p-mTOR, p-AMPK, ERK, and p-ERK. Notably, mTOR and p-mTOR exhibited contrasting expression patterns, while the expression of the other proteins remained consistent following BKS-112 treatment (Figure 6E). Additionally, PI3K expression increased, whereas p-PI3K expression decreased. AKT protein levels remained unchanged, with a slight reduction in p-AKT expression observed after treatment with both BKS-112 and SAHA. These findings illuminate the regulatory patterns of key proteins involved in ROS-mediated processes, providing a comprehensive understanding of the molecular mechanisms affected by BKS-112 in MDA-MB-231 cells shown in Figure 6F.

## 4. Discussion

TNBC is a heterogeneous and recurrent cancer characterized by high metastasis rates, poor prognosis, and limited therapeutic targets. Despite advancements in targeted cancer therapies, effective treatment options for TNBC remain elusive [24]. Clinical trials are currently investigating the efficacy of HDAC inhibitors in treating various malignancies, including head and neck cancer, Hodgkin’s lymphoma, and breast cancer [25,26]. Recent studies have demonstrated the anticancer properties of HDAC inhibitors, which include the inhibition of cell proliferation, colony formation, and migration, as well as the induction of apoptosis, cell cycle alterations, and autophagy. Notably, ACY-1215 (ricolinostat), a well-known HDAC6 inhibitor, has shown efficacy inducing cell proliferation inhibition, apoptosis, and G1 cell cycle arrest, exhibiting significant anti-tumor effects in TNBC cells [27]. In our study, we demonstrated that the suppression of cell survival and proliferation by BKS-112 treatment. Morphological changes and a reduction in colony formation with BKS-112 treatment further underscored its impact on MDA-MB-231 cells. Our findings also revealed that BKS-112 inhibited MDA-MB-231 cell migration, as demonstrated by a wound-healing assay that graphically depicted relative wound density.

BKS-112 regulated α-tubulin acetylation and decreased HDAC6 expression in MDA-MB-231 cells, resulting in reduced tumor growth and invasiveness compared to SAHA. HDAC6 activity assays demonstrated that BKS-112 effectively inhibited HDAC6 expression in a dose-dependent manner. Specifically, BKS-112 increased microtubule acetylation by inhibiting HDAC6, thereby impeding cancer cell migration. In this study, we employed SAHA (vorinostat) as the reference compound for evaluating BKS-112’s anticancer activity. While highly selective HDAC6 inhibitors such as ACY-1215 and tubacin exist, SAHA was chosen as a clinically relevant benchmark for several important reasons. SAHA is an FDA-approved pan-HDAC inhibitor with extensive clinical validation in cancer treatment and well-documented activity in TNBC models, providing a robust therapeutic standard for comparison. Although BKS-112 was rationally designed for HDAC6 selectivity and demonstrates 126-fold selectivity for HDAC6 over HDAC1 (IC_50_ = 42.98 nM for HDAC6 vs. 5432 nM for HDAC1) [23], our Western blot analysis (Figure 2E,F) reveals that BKS-112 also exhibits moderate inhibitory activity against HDAC3 and HDAC4 while sparing HDAC1, HDAC2, and HDAC5. This profile indicates that BKS-112 functions as a moderately selective multi-HDAC inhibitor with preferential HDAC6 activity rather than an exclusively HDAC6-specific inhibitor. In contrast, SAHA causes a broad reduction in HDAC1-7 expression. Therefore, comparing BKS-112 to SAHA allowed us to evaluate whether selective enrichment of HDAC6 inhibition, combined with moderate HDAC3/4 activity, could achieve comparable or superior anticancer efficacy to broad-spectrum HDAC inhibition while potentially reducing toxicity through sparing of class I HDACs critical for normal cellular function. Our results demonstrate that BKS-112 achieves superior effects on colony formation and migration inhibition compared to SAHA (Figure 1E–H), despite its more selective inhibition profile, suggesting therapeutic advantages of this selectivity pattern. The coordinated inhibition of HDAC6, HDAC3, and HDAC4 by BKS-112 may synergistically contribute to its potent anticancer effects, as these isoforms play distinct but complementary roles in cancer cell survival and progression. In the highly invasive TNBC cell line MDA-MB-231, HDAC6 was identified as a critical regulator of cytoskeletal remodeling and cellular migration, as well as a significant contributor to proteolysis within a two-dimensional matrix [22]. Cell cycle arrest at specific checkpoints is a critical mechanism that facilitates cell death [28]. Numerous anticancer agents function through diverse intracellular signaling pathways to inhibit cell proliferation, thereby modulating the cell cycle [29]. There was a decrease in the percentage of cells in the G1 phase, the initial phase of the cell cycle for DNA synthesis and replication. Conversely, there was an increase in the percentage of cells in the G2 phase, suggesting a cellular response to stress, DNA damage, or regulatory mechanisms induced by BKS-112 treatment. The G2 checkpoint primarily regulates the mitosis-promoting functions of cyclin B/CDK1 complex [29]. HDAC inhibitors were shown to elevate the expression of p21, a molecule that inhibits the cyclin–CDK complex, which is essential for cell cycle progression [30]. Furthermore, the maintenance phase of the G2 checkpoint is likely influenced, at least in part, by transcriptional programs regulated by p53.

Our study demonstrates that BKS-112 exhibits anticancer properties by suppressing cyclins and CDKs in MDA-MB-231 cells, which are key regulators of the cell cycle. This downregulation can help overcome resistance mechanisms, rendering cancer cells more susceptible to treatment. In the presence of the HDAC6 inhibitor BKS-112, p21 and p27 bind to cyclins, thereby enhancing the compound’s anticancer effects. This interaction activates p21 and p27, effectively decelerating the cell cycle—an advantageous outcome in cancer therapy aimed at controlling unchecked cell proliferation [31].

A previous study reported that HDAC6 protein is produced, and influences client proteins involved in promoting cellular proliferation, anti-apoptotic signaling, and cell cycle regulation in response to cellular stress [25]. Our findings indicate that BKS-112 significantly increased the population of apoptotic cells in a dose-dependent manner. This compound effectively suppressed cell proliferation, potentially enhancing cancer treatment efficacy by rapidly reducing the cancer cell population. The intrinsic apoptotic pathway modulated by BKS-112 involves the regulation of both pro-apoptotic and anti-apoptotic Bcl-2 family proteins. The tumor suppressor gene p53 plays a central and pivotal role in the complex machinery of apoptosis, a programmed cell death process [31]. In our study, Bax and Bcl-2, serving as facilitators and suppressors of apoptosis, respectively, were critical determinants of cell fate in response to apoptotic stimuli. The increased DAPI fluorescence intensity, indicating chromatin condensation within the nucleus, confirmed the presence of apoptotic nuclei following BKS-112 treatment, underscoring its role in promoting programmed cell death.

Furthermore, it has been reported that HDAC6 plays a protective role in facilitating the clearance of misfolded protein aggregates under conditions of cellular stress, ensuring their elimination within autophagosomes [32]. This process is likely mediated by the ubiquitin-binding protein p62, which has a high binding affinity for LC3 and aids in the removal of protein aggregates [33,34]. In the control group, minimal red fluorescence was observed, indicating the absence of autophagic vacuoles. In contrast, cells treated with BKS-112 exhibited higher red fluorescence intensity, especially at 48 h, reflecting the presence of autophagic vacuoles. BKS-112 treatment led to two significant changes: an increase in LC3 levels, indicating enhanced autophagic activity, and a decrease in p62 levels, signifying the degradation of cellular components during autophagy.

The recruitment of various elements such as autophagy-related genes (ATGs), aggregates, and lysosomes to aggresomes is contingent on two key factors: the structural integrity of the microtubule cytoskeleton and the enzymatic activity of HDAC6, which is responsible for deacetylating tubulin [32]. In the context of BKS-112-induced autophagy in MDA-MB-231 cells, the activities of ATG and Beclin, genes associated with cancer initiation and progression, were heightened. This suggests that BKS-112 treatment activates these genes, potentially facilitating cancer initiation and progression in MDA-MB-231 cells during autophagy-induced cellular stress.

ROS levels are predominantly regulated by cellular metabolism, while ROS simultaneously influence energy metabolism by modulating key metabolic enzymes and signaling pathways involved in oncogenesis [35]. AMP-activated protein kinase (AMPK) is crucial for maintaining redox balance, and NADPH acts as a reducing agent in enzymatic processes while also serving as an antioxidant to counter ROS [36]. HDAC6 is intricately associated with the PI3K/AKT and MAPK/ERK signaling pathways. Inhibiting HDAC6 results in the dephosphorylation of AKT and ERK, thereby suppressing cell proliferation and promoting apoptosis. Additionally, HDAC6 inhibition induces the hyperacetylation of heat shock protein 90, which further reduces the phosphorylation levels of AKT and ERK [37,38]. HDAC6 also prevents the translocation of apoptotic signaling proteins from the cytosol to the mitochondria [39,40]. The regulation of HDAC6 expression activates MAPK pathways, including ERK, JNK, and p38 signaling pathways. These findings suggest that ROS may contribute to the induction of cell death during apoptosis and could play a regulatory role in autophagy through the MAPK signaling pathway [41]. Recent studies have revealed new molecular insights into TNBC progression and treatment resistance. Zhang et al. (2024) demonstrated that the RNA-binding protein POP1 promotes TNBC proliferation through m6A-dependent degradation of CDKN1A mRNA, identifying a potential therapeutic role for the m6A inhibitor STM2457 in suppressing tumor growth [42]. Similarly, Zhang et al. (2025) reported that PRMT1-mediated PARP1 methylation activates the NF-κB (P65) signaling pathway, thereby enhancing lung metastasis and docetaxel resistance [43].

BKS-112 exerts its effects by modulating multiple cellular pathways that are critical to cancer initiation and progression. Mitochondria-generated ROS serve a dual function in cancer, either supporting cell survival or inducing cell death in MDA-MB-231 cells. The PI3K/AKT pathway, essential in cancer biology, influences cell proliferation, metastasis, and drug resistance. The BKS-112 treatment affects key autophagic and apoptotic pathways, including ATG5, PI3K-AKT-mTOR, and MAPK signaling, promoting ROS-mediated apoptosis by altering the Bcl-2/Bax balance and activating cleaved caspase-3. Additionally, BKS-112 triggers mitochondrial apoptosis and autophagy through MAPK pathways, such as ERK, JNK, and p38, in MDA-MB-231 cells. It induces G1 phase cell cycle arrest and causes endoplasmic reticulum stress, leading to caspase-dependent apoptosis. The compound also halts the cell cycle at the G0/G1 phase by stimulating ROS production and inactivating the AKT/CDK/cyclin D1 pathway. These findings suggest a potential role for HDAC6 inhibition in TNBC prevention, with BKS-112 modulating multiple cellular pathways that influence breast cancer cell survival and death mechanisms.

Our choice of SAHA as a reference compound was scientifically justified based on its clinical relevance and BKS-112’s multi-HDAC activity profile. Direct head-to-head comparison with highly selective HDAC6 inhibitors such as ACY-1215 (ricolinostat) or tubacin would provide valuable complementary information. Such comparisons would help delineate whether the anticancer efficacy of BKS-112 results primarily from its potent HDAC6 inhibition or whether the additional moderate activity against HDAC3 and HDAC4 contributes synergistically to its therapeutic effects. Understanding the relative contributions of individual HDAC isoforms to BKS-112’s mechanism of action would facilitate optimization of selectivity profiles for future HDAC inhibitor development. Additionally, comparative studies with other selective HDAC6 inhibitors would better position BKS-112 within the landscape of HDAC-targeted therapies and identify potential advantages in terms of potency, selectivity, pharmacokinetics, or therapeutic index. These comparative evaluations are planned for future investigations to comprehensively characterize BKS-112’s therapeutic positioning for TNBC treatment. Although the current study provides detailed in vitro evidence for the HDAC6-selective inhibition and anticancer activity of BKS-112, certain limitations remain. These include the use of a single TNBC cell line, the absence of in vivo validation, and the lack of pharmacokinetic or toxicological data. Furthermore, potential assay interferences related to compound solubility and cellular heterogeneity cannot be excluded. Future studies will aim to validate BKS-112 across multiple breast cancer subtypes, explore its efficacy and safety in vivo, and investigate its impact on EMT and immune-regulatory pathways.

## 5. Conclusions

This study demonstrated that BKS-112 effectively targets HDAC6 activity, particularly in MDA-MB 231 cells. It exerts significant effects on apoptosis, cell cycle regulation, and autophagy via an intracellular ROS mechanism. The findings of this study strongly suggest that inducing apoptosis and autophagy through the inhibition of HDAC6, as achieved by BKS-112, could be a promising approach for the treatment of TNBC. Furthermore, this study revealed a key mechanism involving the acetylation of AKT by histone acetyl transferases, in which HDAC6 plays a crucial role in maintaining AKT activity. The role of ROS in the acetylation of AKT and the subsequent inhibition of its phosphorylation is highlighted. HDAC6 inhibition promotes AKT acetylation and reduces phosphorylated AKT levels. This study also sheds light on the pivotal roles of PI3K, AKT, mTOR, and AMPK in regulating cellular metabolism, including processes such as protein synthesis, cell survival, autophagy, and cell death. These metabolic pathways exhibit intricate crosstalk and are influenced by oxidative stress and PI3K/AKT/mTOR signaling. These findings provide valuable insights into the potential of BKS-112 as a therapeutic agent for TNBC and underscore the multifaceted relationships among HDAC6, ROS, and various metabolic pathways in cancer.

## Data Availability

The data that supports the findings of this study are available from the corresponding author upon reasonable request.

## Supplementary Materials

The following supporting information can be downloaded at: https://www.mdpi.com/article/10.3390/antiox14111291/s1, Figure S1: MTT cell viability assays in MCF-7 (ER-positive breast cancer); Figure S2: MTT cell viability assays in SKOV3 (ovarian cancer); Figure S3: MTT cell viability assays in MCF-10A (normal breast epithelial) cells; Figure S4: Western blot analyses to assess HDAC6 expression and the inhibitory effects of BKS-112 across a diverse panel of cancer cell lines, including A549 (lung cancer), DU145 (prostate cancer), Caki-1 (renal cell carcinoma), SKOV3 (ovarian cancer), MDA-MB-231 (TNBC), and MCF-7 (ER-positive breast cancer).

## Abbreviations

ATG	Autophagy-related gene
AMPK	Adenosine monophosphate-activated protein kinase
BCA	Bicinchoninic acid
CDKs	Cyclin-Dependent Kinases
DAPI	4′,6-diamidino-2-phenylindole
DCFDA	Dichlorodihydrofluorescein diacetate
DMEM	Dulbecco’s Modified Eagle’s Medium
DPBS	Dulbecco’s phosphate-buffered saline
ERK	Extracellular signal-regulated kinase
FBS	Fetal bovine serum
FITC	Fluorescein isothiocyanate
HDACs	Histone deacetylases
HRP	Horseradish Peroxidase
ICC	Immunocytochemistry
JNK	Jun N-terminal kinase
LC3	Light chain 3
MMPs	Matrix metalloproteinases
MTT	3-(4,5-dimethylthiazol-2-yl)-2,5-diphenyl-2H-tetrazolium bromide
mTOR	Mammalian target of rapamycin
PARP	Poly-ADP ribose polymerase
PI3K	Phosphatidylinositol 3-kinase
PVDF	Polyvinylidene difluoride
SAHA	Suberoylanilide hydroxamic acid
SDS-PAGE	Sodium dodecyl-sulfate polyacrylamide gel electrophoresis
TIMPs	Tissue inhibitors of metalloproteinase
TNBC	Triple-negative Breast Cancer
