# Supplementary figures and images for: BKS-112, a Selective Histone Deacetylase 6 Inhibitor, Suppresses Triple-Negative Breast Cancer Cells via AKT/mTOR Pathway
Source: Antioxidants (Basel). 2025 Oct 28;14(11):1291. doi: 10.3390/antiox14111291 (PMC12649169; doi:10.3390/antiox14111291)

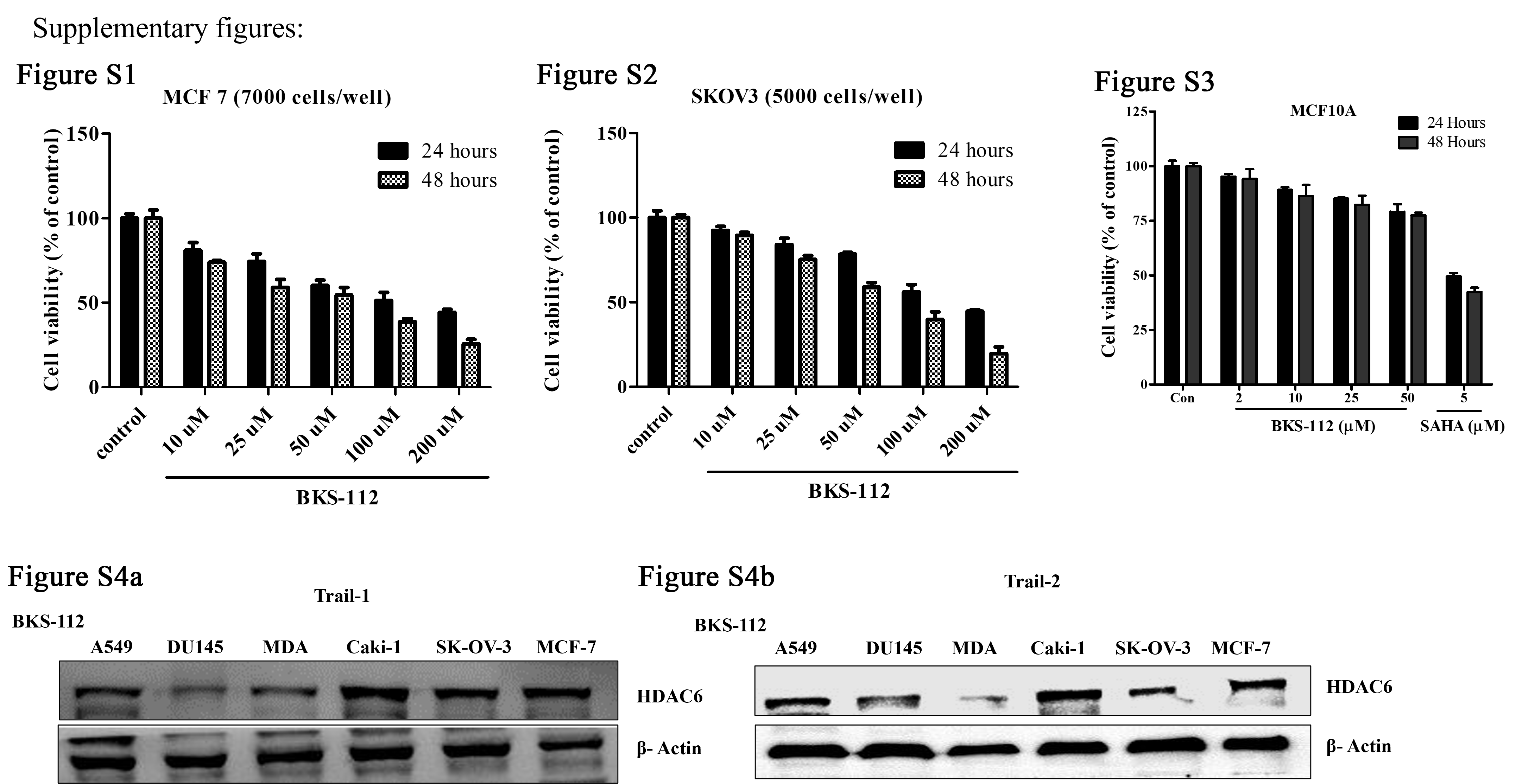

Supplement: Supplementary file 1 [file antioxidants-14-01291-s001.zip › antioxidants-3905284-supplementary.tiff]
